# Association between preoperative cognitive impairment and perioperative neurocognitive disorders within 30 days after surgery in elderly patients: a secondary exploratory retrospective analysis

**DOI:** 10.3389/fragi.2026.1835897

**Published:** 2026-09-07

**Authors:** Keke Song, Ping He, Xiaoqi Zhao, Rongsheng Zhou, Yaomin Zhu, Xiaofei Liu, Xin Zhang

**Affiliations:** 1 Department of Anesthesiology, The First Affiliated Hospital of Xi’an Jiaotong University, Xi’an, Shaanxi, China; 2 Department of Orthopedics, The Second Affiliated Hospital of Fourth Military Medical University (FMMU), Xi’an, Shaanxi, China; 3 Department of Anesthesiology, The Second Affiliated Hospital of Fourth Military Medical University (FMMU), Xi’an, Shaanxi, China; 4 Center for Regenerative and Reconstructive Medicine, Med-X Institute, The First Affiliated Hospital of Xi’an Jiaotong University, Xi’an, China

**Keywords:** cognitive assessment, elderly patients, perioperative neurocognitive disorders, preoperative cognitive performance, secondary exploratory retrospective analysis

## Abstract

**Background:**

Preoperative cognitive impairment (PCI) is a recognized risk factor for postoperative cognitive dysfunction, but its association with the broader syndrome of perioperative neurocognitive disorders (PND) within 30 days after surgery requires further investigation. This study aimed to evaluate the association between preoperative cognitive performance, assessed by the Montreal Cognitive Assessment (MoCA), and the risk of PND in elderly patients.

**Methods:**

This secondary exploratory retrospective analysis based on a completed prospective randomized controlled trial (RCT) cohort included 236 patients aged ≥65 years undergoing non-cardiac surgery under general anesthesia. Preoperative cognitive performance was evaluated using the MoCA, with a score <26 defining PCI. PND was defined as either postoperative delirium (assessed daily with CAM-ICU) or delayed neurocognitive recovery (assessed by MoCA at 7 and 30 days postoperatively). Logistic regression models were used to analyze the association between PCI (as a binary and continuous variable) and PND, adjusting for confounders including age, education, frailty, and surgical factors.

**Results:**

The overall incidence of PND was 40.7%. Patients with PCI (n = 81) had a significantly higher incidence of PND compared to those without PCI (74.1% vs. 23.2%, p < 0.001). In unadjusted analysis, PCI was associated with a 9.44-fold increased odds of PND (OR = 9.44, 95% CI: 5.07–17.58). After multivariable adjustment, PCI remained independently associated with PND (adjusted OR = 6.95, 95% CI: 2.71∼17.79). When analyzed as a continuous variable, each 1-point increase in the MoCA score was associated with a 32% reduction in the odds of PND (adjusted OR = 0.68, 95% CI: 0.58∼0.80). Subgroup analyses showed no statistically significant interaction was observed with age, education, or frailty.

**Conclusion:**

Preoperative cognitive impairment, assessed by the MoCA, is an independent risk factor for PND within 30 days after surgery in elderly patients. Preoperative MoCA screening may help identify high-risk patients for targeted interventions.

## Introduction

Perioperative neurocognitive disorders (PND), encompassing postoperative delirium (POD) and postoperative cognitive dysfunction (POCD), represent a formidable challenge in anesthesiology and perioperative medicine, particularly for the aging surgical population. Following non-cardiac surgery, the incidence of delayed neurocognitive recovery approaches 34.5% at hospital discharge, with POCD persisting in approximately 9.9%–11.7% of patients at 3 months (Monk et al., 2008; Paredes et al., 2016; Moller et al., 1998; Kong et al., 2022). In high-risk cohorts like hip fracture patients, POD incidence can reach up to 65% (Rudolph and Marcantonio, 2011; Evered et al., 2022). These disorders are not transient events; they are robustly associated with devastating long-term outcomes, including accelerated cognitive decline, increased mortality, functional dependency, and substantial healthcare costs, imposing a significant epidemiological and economic burden (Kong et al., 2022; Evered et al., 2022). Consequently, identifying modifiable risk factors and vulnerable patients preoperatively is a critical imperative for improving surgical safety and long-term neurological health.

A principal and well-established predisposing factor for POCD is pre-existing cognitive impairment (Evered et al., 2018; Pas et al., 2022; Granger et al., 2025). Patients with subtle cognitive deficits prior to elective surgery face a markedly higher risk of developing POD and long-term cognitive decline (Granger et al., 2025; Weiss et al., 2023). The prevalence of preoperative cognitive impairment (PCI) is significant, and its assessment is crucial for risk stratification. The Montreal Cognitive Assessment (MoCA) is recognized as a superior instrument for detecting mild cognitive impairment (MCI) in preoperative settings, offering higher sensitivity than the Mini-Mental State Examination (MMSE) (Pas et al., 2022; Nasreddine et al., 2005; Pinto et al., 2019). While brief tools like the Mini-Cog are used, their predictive utility for PND can be inconsistent, and MoCA has demonstrated a better combination of sensitivity and specificity for preoperative cognitive screening (Pas et al., 2022; Fiamanya et al., 2022).

Beyond isolated cognitive deficits, the multidimensional syndrome of frailty has emerged as a potent independent predictor of adverse postoperative outcomes, including PND (Evered et al., 2020; Jin et al., 2023). Frailty, characterized by decreased physiological reserve and resilience, is highly prevalent in surgical populations and is intrinsically linked to vulnerability to stressors (Evered et al., 2022; Jin et al., 2023). The pathophysiological overlap between frailty and neurocognitive decline suggests their potential combined impact on postoperative brain health, making it essential to account for frailty when evaluating the specific contribution of PCI.

However, most prior studies have concentrated on the link between pre-existing cognitive deficits and acute postoperative delirium in isolation, often relying on legacy screening tools like the Mini-Mental State Examination or the Mini-Cog, which suffer from low sensitivity for mild cognitive impairment and notable ceiling effects (Wang et al., 2023). The relationship between objectively measured preoperative cognitive performance—using a highly sensitive tool like the Montreal Cognitive Assessment—and the broader, continuum-based endpoint of perioperative neurocognitive disorders remains underexplored. As defined by the 2018 International Nomenclature Consensus Working Group, perioperative neurocognitive disorders serve as an overarching classification encompassing both the acute, fluctuating disturbances of postoperative delirium and the subacute cognitive decline of delayed neurocognitive recovery diagnosed within 30 days post-surgery (Krishnan et al., 2017).

This study addresses a critical gap in perioperative medicine by evaluating perioperative neurocognitive disorders as a unified 30-day outcome and deploying the Montreal Cognitive Assessment as both a categorical and continuous variable. Establishing a continuous dose-response relationship between baseline scores and the risk of neurocognitive decline fundamentally advances clinical decision-making. It transitions cognitive screening from a binary diagnostic triage assessment into a precision risk-stratification tool, empowering clinicians to implement targeted, risk-adjusted protocols—such as tailored anesthetic depth management, optimized perioperative hemodynamics, and multicomponent non-pharmacological delirium prevention strategies—for highly vulnerable older adults.

## Materials and methods

### Study design and participants

This study is a secondary exploratory retrospective analysis based on a completed prospective randomized controlled trial cohort (ChiCTR1900021720) conducted from March 2019 to December 2020 at The First Affiliated Hospital of Xi’an Jiaotong University. All participants were consecutively recruited according to the eligibility criteria during the study enrolment period. The parent was designed to evaluate dexmedetomidine on blood brain barrier protection and postoperative cognitive dysfunction prevention in elderly surgical patients. The experimental group was dexmedetomidine group, the control group was non dexmedetomidine group. In this study, we selected the RCT control group data for secondary analysis to avoid the effect of dexmedetomidine on postoperative cognition. Meanwhile, we explicitly state in the revised manuscript that the predictive analysis exploring the association between preoperative MoCA-assessed cognitive impairment and postoperative neurocognitive disorders was not pre-specified in the original registered protocol of the parent RCT. This work is entirely a *post hoc* exploratory analysis conducted after the completion of the parent trial. Consequently, the current secondary analysis is subject to the strict inclusion and exclusion criteria of the original trial, which inherently introduces a degree of selection bias and limits the broader generalizability of the findings to unselected, high-risk surgical populations. This approach aligns with the Strengthening the Reporting of Observational Studies in Epidemiology guidelines for the rigorous reporting of secondary observational analyses.

Inclusion criteria included patients 65 years or older undergoing surgery at The First Affiliated Hospital of Xi'an Jiaotong University who underwent non-cardiac, non-transplant, and non-neurological elective surgery with general anesthesia, ASA grade I-III. Patients were excluded if they underwent neurosurgical procedures and could not cooperate to complete clinical scale assessment. The study was approved by the institutional review board (XJTUIAF2017LSL-015), and all patients provided written informed consent. This study has been registered as a clinical trial (ChiCTR1900021720).

### Anesthesia and perioperative care

We monitored the American Society of Anesthesiologists (ASA), SedLine, and end-tidal gas intraoperative. Anesthesia was induced intravenously with sufentanil, lidocaine, etomidate, and rocuronium, maintained with inhaled sevoflurane and intravenous infusion of propofol and remifentanil and none of the patients used dexmedetomidine. During the surgery, the change of blood pressure was within ± 20% of the baseline, the nasopharynx temperature was maintained between 36 °C and 37.5 °C, the end-tidal pressure of carbon dioxide was adjusted to 35–45 mmHg, and the blood glucose was adjusted to 3.9–11.1 mmol/L. By adjusting the dose of anesthetic drugs, the patient safety index (PSI), an index of anesthesia depth, was maintained between 25 and 50. The patient-controlled analgesia pump could provide postoperative analgesia for 72 h, and sufentanil was injected into the pump. Pain relief was achieved by meperidine hydrochloride or nonsteroidal anti-inflammatory drugs 3 days after surgery when needed.

### Data collection

We collected the patient’s detailed medical history and preoperative examination results before surgery. Trained evaluators screened patients for postoperative delirium (POD) twice a day using the Richmond agitation sedation scale (RASS) and the Confusion Assessment Method (CAM) scale preoperatively and 1–7 days postoperatively. If the patient met the diagnostic criteria at any time within 7 days after surgery, we considered that postoperative delirium occurred.

The Montreal Cognitive Assessment Score (MoCA) and Lawton Instrumental Activities of Daily Living (IADL) were assessed preoperatively and 7 and 30 days postoperatively to assess delayed neurocognitive recovery (DNR). PND was considered as POD or DNR within 30 days after surgery. Postoperative complications were defined as those occurring during hospitalization after surgery.

### Neuropsychological assessment

POD was evaluated using the standardized CAM scale. The CAM scale was based on the following four basic characteristics of delirium: (1) acute onset and fluctuating course; (2) inattention; (3) disorganized thinking; and (4) altered level of consciousness. According to CAM, a diagnosis of delirium requires the presence of features 1, 2, and either 3 or 4.

DNR was evaluated using the MoCA scale. To minimize the learning effect associated with repeated administration of the MoCA, we recruited some control subjects who did not undergo surgery. These control subjects were community volunteers aged 65 or above, and their inclusion and exclusion criteria were the same as surgical patients. In the present study, the evaluation date of volunteers and the evaluation date of surgical patients overlap. These volunteers were recruited for our cognitive study. The difference between postoperative and preoperative test scores was defined as the learning effect. The Z score for each individual was calculated and compared with baseline scores and divided by the standard deviation (SD) of the learning effect. DNR was diagnosed when the Z score was greater than 1.96.

PND was considered as POD or DNR occurring within 30 days after surgery. This composite endpoint aligns with the recommendations of the International Nomenclature Consensus Working Group, which proposes “perioperative neurocognitive disorders” as an overarching term that includes both delirium (POD) and cognitive decline up to 30 days postoperatively DNR. This approach facilitates a comprehensive assessment of clinically significant neurocognitive morbidity in the acute to subacute postoperative period.

All neuropsychological functions were tested 1 day before the surgery and on postoperative 7 and 30 days in person or by phone.

### Frailty assessment

Frailty was determined using the Fried Frailty Phenotype. Participants were classified as frail if they met ≥3 of the five criteria: unintentional weight loss, self-reported exhaustion, low grip strength, slow walking speed, and low physical activity. Assessments were performed within 24 h before surgery.

### Statistical analysis

The study sample was divided into two subgroups based on preoperative MoCA scores.

A fixed cutoff of <26 was used to define PCI without education adjustment; patients scoring ≥26 were classified as having no cognitive impairment, and those scoring <26 as having cognitive impairment. Missing data were processed by multiple imputation. The baseline characteristics of all patients were stratified according to these two groups; continuous variables were presented as mean ± standard deviation (SD) or median and interquartile range (IQR). Categorical data were expressed as numbers or percentages and were compared using the chi-squared test. Summaries of the categorical data are indicated as the percentage or number. The Lowess smoothing method was employed to explore the association of MoCA scores and PND. To facilitate the clinical interpretation of our findings, we employed the logistic regression models to assess risk factors related to PND. Results were presented as odds ratios (ORs) with 95% confidence intervals (CIs). The data were analyzed using the R Statistical Software (http://www.R-project.org, The R Foundation) and Free Statistics software versions 2.2. A two-sided P-value <0.05 was statistically significant.

Missing data were scattered across several secondary laboratory covariates with an overall missing proportion less than 3%, while key predictors (MoCA scores, frailty) and PND outcome were fully recorded without missing values. Missingness was judged to be missing completely at random. The primary multivariable regression was performed via complete case analysis. Multiple imputation was conducted as a sensitivity analysis to assess the robustness of findings. Results derived from complete cases and pooled imputed datasets were compared mutually.

## Results

### Patient characteristics

A total of 236 patients were analyzed in the study, including no cognitive impairment (n = 155) and cognitive impairment (n = 81). The baseline characteristics of no cognitive impairment and cognitive impairment are presented in Table 1. The average age of the patients was 71.5 ± 5.0 years, and 69.1% were male. Gastrointestinal surgery (19.9%), other abdominal procedure (34.7%) and major urologic surgery (26.7%) were the common surgical procedures. The average anesthesia duration was 199.2 ± 118.7 min. The incidence of PND is 40.7%.

**TABLE 1:** Baseline characteristics of patients.

Variables	Overall (N = 236)	No cognitive impairment (n = 155)	Cognitive impairment (n = 81)	*p* value
Age, years	71.5 ± 5.0	70.1 ± 4.0	74.3 ± 5.4	<0.001
Male sex	163 (69.1%)	108 (69.7%)	55 (67.9%)	0.779
Body mass index, kg/m^2^	23.5 ± 3.2	23.7 ± 3.3	23.2 ± 3.1	0.274
Education, years	11.2 ± 3.6	11.6 ± 3.2	10.5 ± 4.1	0.022
Hypertension	100 (42.4%)	56 (36.1%)	44 (54.3%)	0.007
Diabetes	54 (22.9%)	25 (16.1%)	29 (35.8%)	<0.001
Coronary heart disease	30 (12.7%)	20 (12.9%)	10 (12.3%)	0.903
COPD	6 (2.5%)	1 (0.6%)	5 (6.2%)	0.019
Chronic kidney disease	21 (8.9%)	12 (7.7%)	9 (11.1%)	0.388
Frailty	65 (27.5%)	36 (23.2%)	29 (35.8%)	0.04
Anaemia	54 (22.9%)	25 (16.1%)	29 (35.8%)	<0.001
Hypoalbuminemia	34 (14.4%)	15 (9.7%)	19 (23.5%)	0.004
Albumin	40.0 ± 4.7	40.9 ± 4.6	38.2 ± 4.4	<0.001
MoCA	25.2 ± 3.3	27.3 ± 1.1	21.0 ± 1.7	<0.001
IADL	47 (19.9)	28 (18.1)	19 (23.5)	0.325
ASA	​	​	​	<0.001
I	2 (0.8%)	1 (0.6%)	1 (1.2%)	​
II	204 (86.4%)	146 (94.2%)	58 (71.6%)	​
III	30 (12.7%)	8 (5.2%)	22 (27.2%)	​
Type of surgery	​	​	​	0.122
Major gynecological surgery	12 (5.1%)	8 (5.2%)	4 (4.9%)	​
Major urologic surgery	63 (26.7%)	46 (29.7%)	17 (21%)	​
Gastrointestinal surgery	47 (19.9%)	24 (15.5%)	23 (28.4%)	​
Lung surgery	22 (9.3%)	18 (11.6%)	4 (4.9%)	​
Other abdominal procedure	82 (34.7%)	53 (34.2%)	29 (35.8%)	​
Orthopedics surgery	10 (4.2%)	6 (3.9%)	4 (4.9%)	​
Anesthesia duration	199.2 ± 118.7	183.1 ± 105.8	230.0 ± 135.6	0.004
Blood loss	50.0 (20.0, 200.0)	50.0 (10.0, 175.0)	100.0 (50.0, 200.0)	0.054
Allogeneic blood transfusion	0.0 (0.0, 0.0)	0.0 (0.0, 0.0)	0.0 (0.0, 0.0)	0.015
Atropine use	13 (5.5%)	5 (3.2%)	8 (9.9%)	0.066
Penehyclidine use	225 (95.3%)	149 (96.1%)	76 (93.8%)	0.518
Midazolam use	193 (81.8%)	122 (78.7%)	71 (87.7%)	0.091
Sevoflurane dosage	31.0 ± 19.5	30.5 ± 18.9	32.0 ± 20.5	0.584
Long duration (≥5min) of hypotension	15 (6.4%)	10 (6.5%)	5 (6.2%)	0.934
POD	37 (15.7%)	2 (1.3%)	35 (43.2%)	<0.001
DNR	72 (30.5%)	33 (21.3%)	39 (48.1%)	<0.001
POD and DNR	20 (8.5%)	0 (0)	20 (24.7%)	<0.001
PND	96 (40.7%)	36 (23.2%)	60 (74.1%)	<0.001
Length of hospitalization	13.1 ± 7.6	12.0 ± 6.7	15.1 ± 8.7	0.003
Postoperative hospitalization days	6.0 (3.0, 9.0)	5.0 (3.0, 8.0)	7.0 (4.0, 10.0)	0.008
Postoperative complications	10 (4.2%)	2 (1.3%)	8 (9.9%)	0.004
30 days for readmission	4 (1.7%)	2 (1.3%)	2 (2.5%)	0.609

Patients with preoperative cognitive impairment, compared with those without cognitive impairment, were older (p < 0.001) and more frail (p = 0.04), and had a higher prevalence of anemia and hypoalbuminemia (Table 1). Patients with cognitive impairment had lower years of education and MoCA scores, the average MoCA score was 21.0 ± 1.7, compared with 27.3 ± 1.1 in patients without impairment (p < 0.001). In addition, patients with cognitive impairment experienced longer anesthesia durations, were more likely to receive allogeneic blood transfusions (Table 1). Furthermore, the incidence of POD, DNR, PND respectively was 43.2%, 48.1%, 74.1% in patients with preoperative cognitive impairment, which were significantly higher than patients with no cognitive impairment (respectively p < 0.001), and had longer length of hospitalization (p = 0.003), postoperative hospitalization days (p = 0.008) (Table 1).

### Association between preoperative cognitive performance and PND

The association between preoperative cognitive performance and PND was examined using logistic regression. Confounding is an important issue in multivariate analyses. We selected these confounders on the basis of clinical constraints, p < 0.05 in univariate analysis (Supplementary Table S1) or a change in effect estimate of more than 10%.

We initially included preoperative cognitive performance as a binary variable in the logistic regression model. In the unadjusted model, incidence of PND in patients with preoperative cognitive impairment was 9.44 times higher than patients with no cognitive impairment (OR = 9.44; 95% CI: 5.07∼17.58, p < 0.001) (Table 2). After adjusting for confounding factors in Model 1 (age, education), Model 2 (age, education, albumin, frailty, type of Surgery, anesthesia duration, blood loss), and Model 3 (age, education, albumin, frailty, type of Surgery, anesthesia duration, blood loss, postoperative hospitalization days, length of hospitalization), preoperative cognitive impairment remained independently associated with a higher risk of PND (all p values < 0.001; Table 2).

**TABLE 2:** Multivariable logistic regression analyses of association between preoperative cognitive performance and perioperative neurocognitive disorders.

Model	OR (95%CI)	*P* value
Cognitive performance as a binary variable (cognitive impairment, yes vs. no)		
Unadjusted	9.44 (5.07∼17.58)	<0.001
Model 1	4.95 (2.47∼9.95)	<0.001
Model 2	6.92 (2.70∼17.66)	<0.001
Model 3	6.95 (2.71∼17.79)	<0.001
Cognitive performance as a continuous variable (per point increase in MoCA)		
Unadjusted	0.67 (0.60∼0.74)	<0.001
Model 1	0.74 (0.65∼0.83)	<0.001
Model 2	0.69 (0.59∼0.81)	<0.001
Model 3	0.68 (0.58∼0.80)	<0.001

Unadjusted model adjusted for: none.

Model 1 adjusted for: age, education.

Model 2 adjusted for: age, education, albumin, frailty, type of surgery, anesthesia duration, blood loss.

Model 3 adjusted for: age, education, albumin, frailty, type of surgery, anesthesia duration, blood loss, postoperative hospitalization days, length of hospitalization.

**FIGURE 1:**
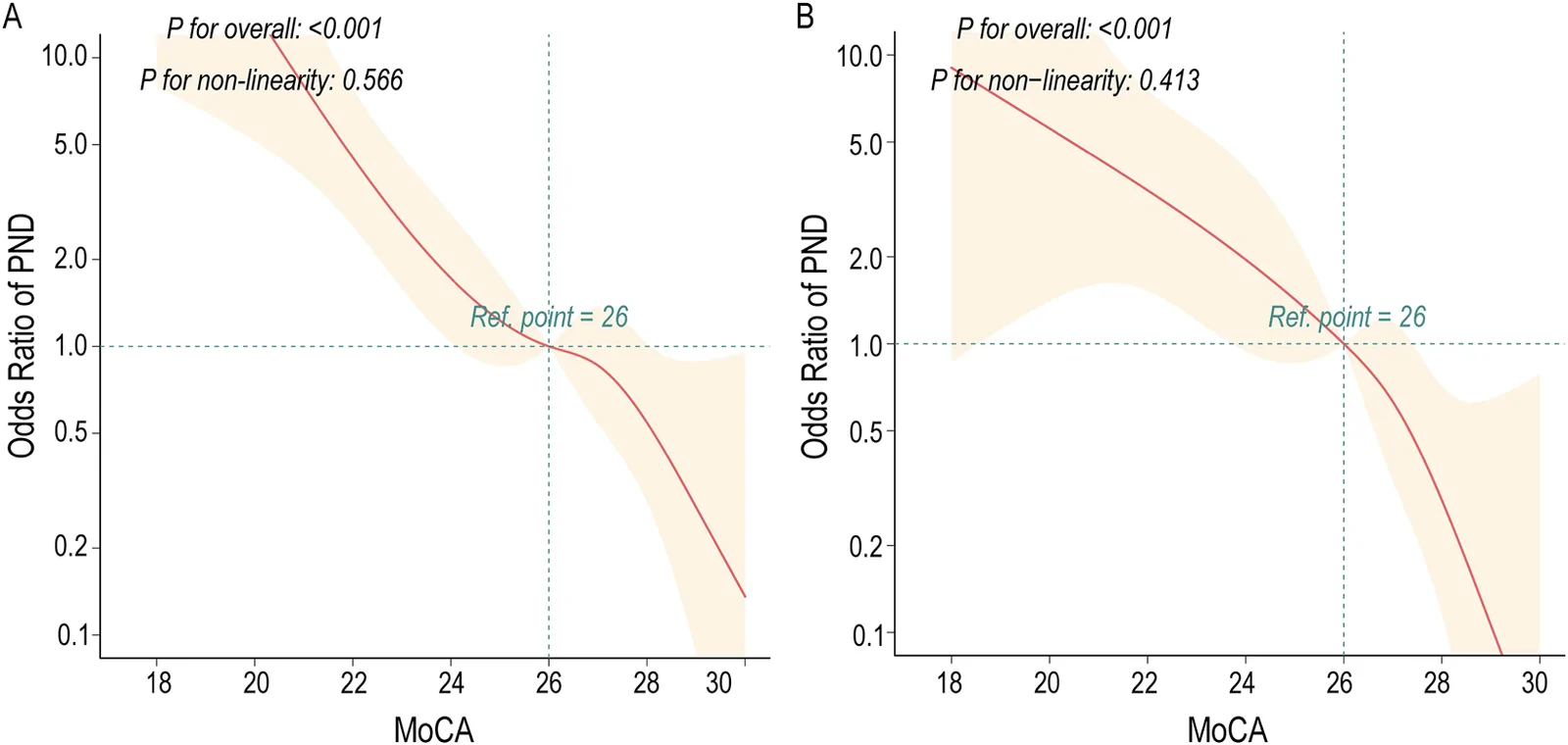
Restricted cubic spline curves depicting the association between MoCA scores and perioperative neurocognitive disorders. **(A)** Unadjusted curves. **(B)** Adjusted curves.

We then examined preoperative cognitive performance as a continuous variable (MoCA scores) in the logistic regression model. In the unadjusted model, with every one point increase in MoCA, the incidence of PND in patients with preoperative cognitive impairment reduced by 33%. Across in all three models (all p values < 0.001; Table 2), the results demonstrated that preoperative MoCA scores were negatively associated with the risk of PND. To illustrate the association between preoperative MoCA scores and PND, we employed restricted cubic splines, whether in non-adjusted model or adjusted model, we observed a linear negative correlation between the two variables (Figure 1, p for non-linearity >0.05). We also conducted a sensitivity analysis by propensity score analyses, including propensity score matching (PSM) and inverse probability treatment weighting (IPTW). In Supplementary Figure S1, the propensity score ROC curve demonstrated an AUC of 83.4%, indicating good discrimination ability of the model in predicting the probability of PCI based on the measured preoperative covariates. Supplementary Figure S2 is propensity score SMD. Plot. The PSM and IPTW results all showed preoperative cognitive impairment remained independently associated with a higher risk of PND (OR = 2.214; 95% CI: 1.003∼4.888, p = 0.0491 and OR = 5.234; 95% CI: 2.927∼9.361, p < 0.001) (Supplementary Table S1).

### Subgroup analyses

**FIGURE 2:**
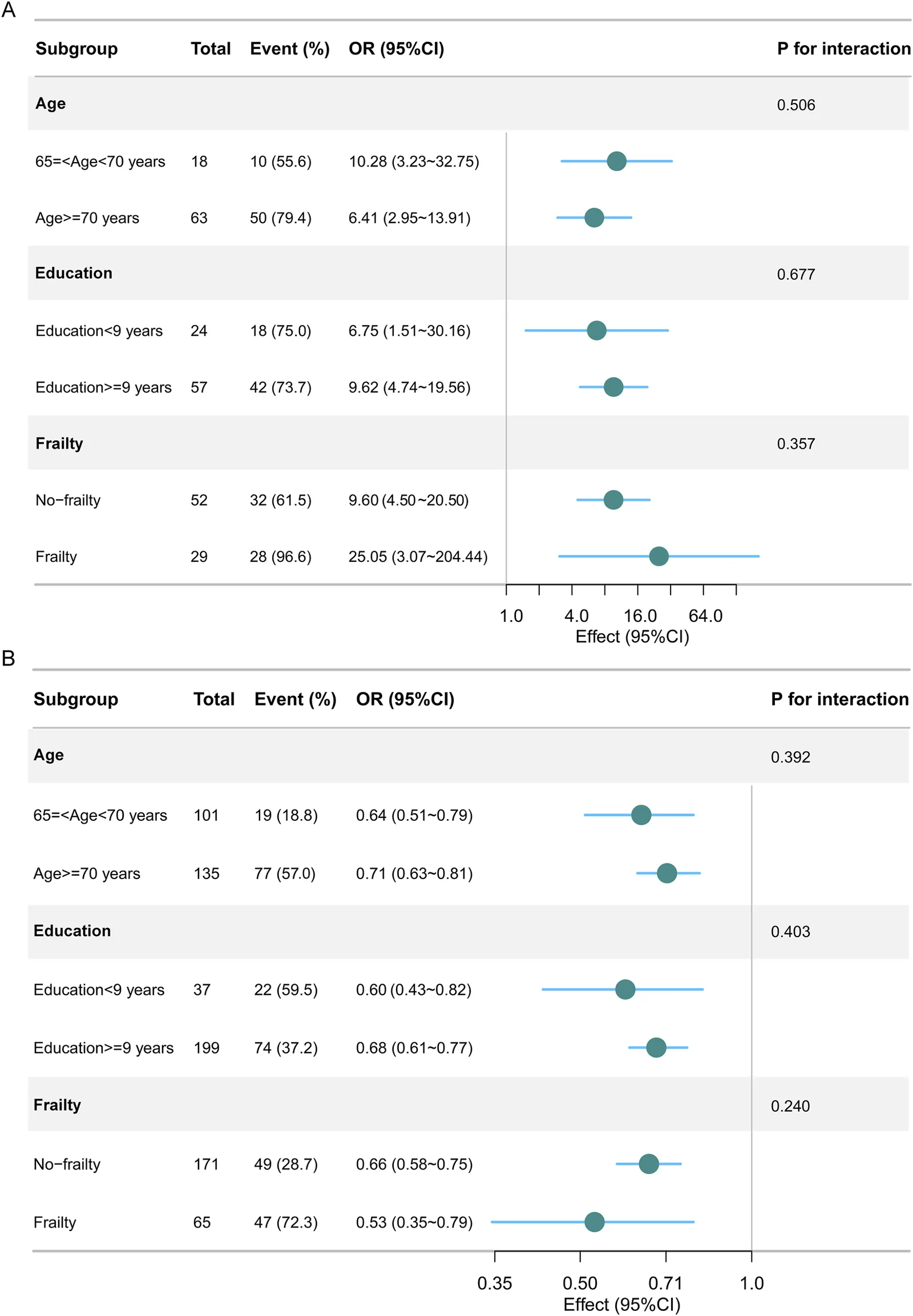
Subgroup analysis of cognitive performance as a binary variable for cognitive impairment patients **(A)** and subgroup analysis of cognitive performance as a continuous variable **(B)**.

Subgroup analyses were carried out to investigate the relationship of age, education and frailty with PND (Figure 2). In Figure 2A, where cognitive performance was analyzed as a binary variable, the effect sizes of each subgroup on the odds of PND in patients with preoperative cognitive impairment remained robust and reliable. No statistically significant interactions were observed with age, education, or frailty (P for interaction: 0.506, 0.677, and 0.357, respectively). In Figure 2B, where cognitive performance was treated as a continuous variable, no statistically significant interactions were observed across these subgroups (P for interaction: 0.392, 0.403, and 0.24, respectively). Preoperative MoCA scores remained negatively associated with the risk of PND in each subgroup.

Sensitivity analysis demonstrated that the association between preoperative cognitive impairment and 30-day PND was consistent in direction, magnitude and statistical significance between complete case analysis and multiple imputation analysis, indicating that minor missing covariate data exerted no meaningful influence on our primary conclusions.

## Discussion

Our study revealed that MoCA score showed a linear association with PND within 30 days after surgery in elderly patients. After multivariate logistic regressions were adjusted for other significant variables, we found that PCI remained independently associated with PND, per point increase in MoCA, the incidence of PND reduced by 32%.

The original design and validation of MoCA are targeted at mild cognitive impairment (MCI), which can identify the gray area between normal aging and early dementia, effectively distinguishing between normal aging and MCI (Nasreddine et al., 2005; Rossetti et al., 2011). Multiple studies have shown that when the cutoff value of MoCA is 26, its sensitivity in detecting MCI in PND is the highest, reaching up to 90%–96% (Kong et al., 2022; Carson et al., 2018). In our present study, PCI was assessed with MoCA test and defined at a score <26. MMSE is primarily used for screening dementia and assessing its severity, with a low sensitivity for MCI (approximately 18%–25%). It is used to evaluate the severity and follow-up of patients with known dementia (Hoops et al., 2009). Meanwhile, multiple comparative studies have shown that MoCA has better detectability for MCI compared to MMSE (Pinto et al., 2019; Jia et al., 2021; Mian et al., 2024). Mini-Cog is a rapid screening tool for dementia in primary care, serving as a minimalist screening tool with very low sensitivity for MCI, its main design goal is to identify dementia, not MCI (Borson et al., 2000). Mariska et al. conducted a screening of MCI in preoperative patients and found that among Mini-Cog, MoCA, MMSE2, O3DY, AD8, SAGE, SLUMS, TICS(-M), QMCI, and Mini-ACE, MoCA had the highest combination of sensitivity and specificity, making it a feasible and effective routine screening for preoperative cognitive function (Pas et al., 2022). Therefore, MoCA is used for the screening of PCI in our present study.

The multivariable regression analysis we conducted minimized the impact of confounding factors on the results. We also performed a subgroup analysis, stratifying the results based on age, years of education and preoperative frailty. Previous studies have indicated that age is the most significant risk factor for PND, with an increased incidence of PND as age increases (Kubota et al., 2018; Chen et al., 2022). Therefore, we selected elderly patients aged 65 years and above, using 70 years as a cutoff. PCI was positively associated with PND in both the 65–69-year and ≥70-year age groups. The results indicate that PCI, as a risk factor for PND, has a universal impact on the elderly patients, emphasizing the significance of identifying PCI in preoperative evaluation for predicting and preventing PND. However, age alone cannot reflect a patient’s overall physiological reserve. Frailty, characterized by decreased physiological reserve and resilience, can more comprehensively capture vulnerability to stressors than chronological age (Evered et al., 2022; Ma et al., 2019).

In our cohort, frailty itself was associated with a higher incidence of PND, consistent with prior evidence that preoperative frailty predicts postoperative neurocognitive disorders (Evered et al., 2022; Evered et al., 2020; Anjaleekrishna et al., 2025). In the frailty subgroup analysis, the effect estimate for PCI appeared larger among frail participants; however, this finding should be interpreted with substantial caution. The frailty subgroup comprised only 29 patients with 28 PND events, yielding an odds ratio of 25.05 with a very wide confidence interval (95% CI: 3.07–204.44). This interval width signals considerable statistical instability and suggests near-complete separation, precluding reliable inference. Furthermore, the P value for interaction was 0.357, which does not establish the absence of effect modification by frailty given the marked disparity in stratum estimates and the limited statistical power. Therefore, while our data are compatible with a synergistic relationship between frailty and PCI, we cannot conclude that frailty modifies the association between PCI and PND. Future adequately powered studies are needed to clarify whether frailty amplifies the risk attributable to PCI.

A retrospective observational study on total hip arthroplasty in elderly patients revealed a significant correlation between preoperative frailty and cognitive decline at 3 and 12 months postoperatively (Evered et al., 2020). The pooled results from another systematic review and meta-analysis, encompassing 16 cohort studies involving a total of 4,805 elderly patients, also unequivocally support the substantial influence of preoperative frailty on the occurrence of PND in elderly surgical patients (Zhao et al., 2024). A large cohort study on elderly patients clearly indicates that patients with preoperative memory impairment have a 2.048-fold higher risk of developing PND 3 months postoperatively compared to patients with normal preoperative cognition (Granger et al., 2025). The study by Wang et al. focused on the potential mechanism of further deterioration of cognitive function in elderly patients with PCI after undergoing anesthesia and surgical procedures, which involves the interaction of the microbiota-gut-brain axis, these phenomena indicate that perioperative stress and inflammatory response exacerbate intestinal flora disruption, intestinal barrier dysfunction, and inflammatory states in the blood of elderly patients with preclinical Alzheimer’s disease, ultimately leading to a decline in cognitive function (Liu et al., 2022). Possible common mechanisms include undiagnosed neurodegenerative diseases, inflammation and the resulting neuroinflammation, neuronal damage, as well as comorbid systemic diseases (Evered et al., 2022; Jin et al., 2023; Anjaleekrishna et al., 2025).

In this study, the incidence of POD in patients with normal preoperative cognition was 1.3%, and the possible reasons were: First, our study exclusively included patients undergoing elective surgery with rigorous preoperative cognitive screening (MoCA > 26), which likely excluded patients with subclinical cognitive impairment who are at higher risk for POD. Second, all patients in our cohort received a standardized perioperative protocol including short-acting anesthetic agents, appropriate depth of anesthesia, multimodal analgesia, which have been shown to reduce POD incidence (Sun et al., 2024; Sumner et al., 2023; Radtke et al., 2013). Third, the relatively small sample size and limited POD events reduced the precision of incidence estimates across subgroups. The observed low POD rate among patients with intact preoperative cognition may be affected by random sampling fluctuation. Larger multicenter cohorts are required to verify this finding.

Several limitations of this study should be noted. First, this single-center secondary analysis was derived from a parent RCT with strict inclusion criteria. We excluded ASA IV patients who could not complete standardized preoperative cognitive assessments due to unstable physical conditions, alongside cardiac, transplant and neurosurgical cases. The cohort was predominantly male (69.1%), and PND outcomes were only tracked for 30 days postoperatively. These factors collectively restrict the generalizability of our findings to higher-risk patients, specialized surgical populations and individuals with long-term cognitive changes. Second, insufficient statistical power limited our subgroup analyses. Despite markedly disparate OR estimates for PND across frail and non-frail participants (25.05 vs. 9.6), the interaction P value was 0.357. A non-significant interaction does not rule out effect modification by frailty, and wide unstable confidence intervals in the frailty subgroup reflect limited PND events (n = 96). Finally, the present predictive analysis was not pre-specified in the registered protocol of the parent RCT, which represents an inherent limitation of this *post hoc* exploratory investigation. Large multicenter prospective studies with balanced gender distribution, broader enrolment criteria and extended follow-up are required to validate our conclusions. Preoperative cognitive impairment is a key risk factor for predicting the occurrence of PND, this provides a strong basis for early identification of high-risk groups in clinical settings and implementation of precise interventions.

In conclusion, our findings demonstrate a strong and independent association between preoperative cognitive impairment, as assessed by the MoCA, and an increased risk of perioperative neurocognitive disorders within 30 days after non-cardiac surgery in elderly patients. This association exhibits a dose-response relationship. Although we adjusted for a comprehensive set of clinically relevant covariates, including frailty, the observational nature of this study precludes definitive causal inference. Residual confounding by unmeasured factors (e.g., subclinical neurodegenerative pathology, genetic predisposition, or detailed socioeconomic status) may persist. Nevertheless, our results reinforce the critical importance of preoperative cognitive screening using sensitive tools like the MoCA.

## Conclusion

Preoperative cognitive impairment, assessed by the MoCA, is independently associated with a significantly higher risk of PND within 30 days after surgery in elderly patients. Preoperative MoCA screening may help identify high-risk patients who could benefit from targeted perioperative interventions and closer monitoring. Further prospective studies with large samples and multiple centers are needed to determine the causality and evaluate whether interventions based on preoperative MOCA screening can effectively reduce the incidence of PND.

## Data Availability

The raw data supporting the conclusions of this article will be made available by the authors, without undue reservation.
