# Supplementary material for: Association between preoperative cognitive impairment and perioperative neurocognitive disorders within 30 days after surgery in elderly patients: a secondary exploratory retrospective analysis
Source: Front Aging. 2026 Sep 7;7:1835897. doi: 10.3389/fragi.2026.1835897 (PMC13590751; doi:10.3389/fragi.2026.1835897)
Supplement: Supplementary file 1 [file Supplementaryfile1.docx]

**Supplementary files**

**Figure S1** Flowchart

**S-****Table 1** Univariate analysis for the odds of perioperative neurocognitive disorders.

| **Variable** | **OR_95%CI** | ***P*-value** |
| --- | --- | --- |
| Age, years | 1.28 (1.19~1.37) | <0.001 |
| Male | 0.83 (0.47~1.45) | 0.509 |
| BMI, kg/m2 | 0.92 (0.85~1) | 0.053 |
| Education, years | 0.94 (0.87~1.01) | 0.08 |
| Hypertension | 1.36 (0.81~2.31) | 0.247 |
| Diabetes | 1.35 (0.73~2.49) | 0.339 |
| Coronary Heart Disease | 1.32 (0.61~2.86) | 0.476 |
| COPD | 3 (0.54~16.72) | 0.21 |
| Chronic kidney disease | 0.71 (0.27~1.82) | 0.475 |
| Anaemia | 2.68 (1.44~5) | 0.002 |
| Hypoalbuminemia | 2.74 (1.29~5.78) | 0.008 |
| Albumin | 0.9 (0.85~0.96) | 0.001 |
| frailty | 6.5 (3.44~12.29) | <0.001 |
| MoCA | 0.67 (0.6~0.74) | <0.001 |
| MoCA≤26 | 9.44 (5.07~17.58) | <0.001 |
| mini-Cog score | 0.39 (0.29~0.53) | <0.001 |
| IADL | 6.12 (2.97~12.61) | <0.001 |
| ASA II | 0.59 (0.04~9.63) | 0.714 |
| ASA III | 1.73 (0.1~30.45) | 0.709 |
| Major urologic surgery | 1.3 (0.25~6.68) | 0.753 |
| Gastrointestinal surgery | 6.75 (1.33~34.27) | 0.021 |
| Lung surgery | 2.86 (0.5~16.43) | 0.239 |
| Other abdominal procedure | 4.53 (0.94~21.99) | 0.061 |
| Orthopedics surgery | 11.67 (1.53~89.12) | 0.018 |
| Anesthesia duration | 1.01 (1~1.01) | <0.001 |
| Long duration (≥5min) of hypotension | 0.97 (0.33~2.82) | 0.956 |
| Blood loss | 1 (1~1) | 0.001 |
| Allogeneic blood transfusion | 1 (1~1) | 0.027 |
| Atropine use | 5.31 (1.42~19.84) | 0.013 |
| Penehyclidine use | 0.56 (0.16~1.88) | 0.344 |
| Midazolam use | 1.35 (0.68~2.69) | 0.393 |
| Sevoflura dosage | 1.02 (1.01~1.04) | 0.004 |
| Length of hospitalization | 1.1 (1.06~1.15) | <0.001 |
| Postoperative hospitalization days | 1.17 (1.1~1.26) | <0.001 |
| Postoperative complications | 6.27 (1.3~30.22) | 0.022 |
| 30 days for readmission | 1.47 (0.2~10.61) | 0.704 |

**S-Figure1**. Propensity score ROC curve

**S-Figure2**. Propensity score SMD.Plot.

Association between preoperative cognitive performance and PND with crude analysis, multivariable analysis and propensity score analyses.

| Models | OR (95%CI) | P value |
| --- | --- | --- |
| Unmatched.crude | 9.444 (5.074~17.579) | <0.001 |
| Multivariable.adjusted | 5.839 (2.271~15.011) | <0.001 |
| PropensityScore.adjusted | 3.243 (1.558~6.753) | 0.0017 |
| PropensityScore.Matched | 2.214 (1.003~4.888) | 0.0491 |
| Weighted.IPTW | 5.234 (2.927~9.361) | <0.001 |
